# Evidence of ritual breakage of a ground stone tool at the Late Natufian site of Hilazon Tachtit cave (12,000 years ago)

**DOI:** 10.1371/journal.pone.0223370

**Published:** 2019-10-16

**Authors:** Laure Dubreuil, Ahiad Ovadia, Ruth Shahack-Gross, Leore Grosman

**Affiliations:** 1 Department of Anthropology, Trent University, Peterborough, Canada; 2 The Israel Museum, Jerusalem, Israel; 3 Institute of Archaeology, The Hebrew University, Jerusalem, Israel; 4 Department of Maritime Civilizations, Recanati Institute for Maritime Studies, Leon H. Charney School for Marine Sciences, University of Haifa, Haifa, Israel; 5 The Jack, Joseph and Morton Scholion-Mandel School for Advanced Studies in the Humanities, The Hebrew University of Jerusalem, Jerusalem, Israel; Max Planck Institute for the Science of Human History, GERMANY

## Abstract

Destruction of valuables is a common behavior in human history. Ethnographic data show the polysemic, but fundamentally symbolic, nature of this act. Yet, research aimed at exploring symbolic destruction in prehistoric societies has underlined the difficulties in establishing unambiguous evidence for such behaviour. We present here the analysis of a basalt tool fragment which provides evidence for intentional breakage associated with ritual activity 12,000 years ago. The tool fragment was part of a unique assemblage of grave goods deposited in a burial pit of a woman suggested to have been a shaman (Hilazon Tachtit cave, Southern Levant). The reconstruction of the artefact’s life history through morphological, 3D, use wear, residue and contextual analyses suggest that: 1) the fragment was initially part of a shallow bowl used for mixing ash or lime with water; 2) the bowl was subsequently intentionally broken through flaking along multiple axes; 3) The bowl was not used after its breakage but placed in a cache before the interment of the deceased, accompanied with other special items. The broken bowl fragment underlines the ritualistic nature of the act of breakage in the Natufian society. The research presented in this paper provides an important window into Natufian ritual behaviour during the critical period of transformation to agricultural communities. In addition, our results offer new insight into practices related to intentional destruction of valuables associated with death-related ceremonies at the end of the Palaeolithic.

## Introduction

Destruction of valuables is a common behavior in human societies (e.g., [1–22]). The potlatch feasts of the West Coast tribes of North America are a classic example of such a practice, where goods and valuables are distributed and occasionally destroyed [1–5, 13–14]. In this context, destruction may be viewed as part of a custom aiming at maintaining, negotiating and validating tribal or intertribal social organization (e.g., [1, 13], but see [23]). However, in ethnographic reports, the most common event where destruction occurs is linked to death rituals (e.g., [8, 12, 15, 19, 20, 22]), in various forms determined, primarily, by religious beliefs.

In the present study, we provide early evidence of symbolic destructive behaviour during the Palaeolithic, grounded in a detailed analysis of a unique artifact found in a sealed grave at Hilazon Tachtit cave (Southern Levant). The grave itself is dated to 12,000 calBP and was suggested to be a shaman burial reflecting complex ritual activities [24]. Here we show that intentional breakage was also part of these activities.

Several occurrences of ritual destruction have been suggested for the Paleolithic period (e.g., [25–31]). The earliest evidence, dated to about 26,000 BP, comes from Dolni Věstonice (Czech Republic), in the form of the making and ‘exploding’ of clay figurines [25, 32–33]. According to Vandiver et al. [25], the figurines were broken through thermal shock, requiring intentional effort and practice. Fragmented figurines were predominantly found in kilns and ash lenses, on settlement fringes, suggesting the special and non-utilitarian nature of the behaviour [25]. Deliberate, potentially symbolic, destruction of a hut through burning has also been suggested at Kharaneh IV (Jordan), dated to around 19,000 calBP [26].

Other early evidence of intentional destruction, this time of stone objects, was proposed for the Paleolithic period in the Levant, associated more specifically with the Kebaran (23,000–17,500 calBP) at Ohalo II [27] and the Geometric Kebaran (17,500–14. 600 calBP) at Neve David, in this site related to burial practices [28]. These hypotheses remain, however, to be fully investigated. Similar claims have been made for the Natufian culture (15,000–11,500 calBP; e.g., [29, 30]). Likewise, at Arene Candide (Italy) detailed analyses of ochre-painted pebbles dated to about 11,000 calBP concluded that they were intentionally fragmented [31]. These pebbles are described as being similar to others found associated with burials during previous excavations at the site.

Instances of specific treatment of fragmented items found in the Levantine Epipaleolithic are worth mentioning here. At Wadi Hammeh 27 (ca. 12,000–12,500 calBP, Jordan), large decorated slab fragments were placed in an arrangement after the breakage of the slabs, in a likely symbolic or ritual context [34]. Re-use and specific discard of fragments of decorated pieces is also observed at the same site [35– 36]. In general, recycling of large tool fragments, such as those of grinding slabs or mortars, is commonly observed in construction in Natufian sites. Scholars have suggested that this act of recycling was neither ‘practical’ nor mundane but symbolic in essence ([37], see also [38] for later period).

Ethnographic accounts report that people choose various materials and techniques for intentional destruction of objects, including burning, breakage, drilling, and throwing away [6–12,14,15, 20]. Alternatively, objects may be buried [12,15, 20]. Unsurprisingly, several of these practices, as throwing away, are difficult to detect in the archaeological record. Keeping this caveat in mind, this paper focuses on intentional breakage of objects through the analysis of a fragment derived from a ground stone tool (GST).

GSTs are stone implements such as grinding or pounding tools, abraders, percussive tools and stone vessels used or manufactured by percussion and abrasion [39]. Intentional destruction has been often addressed in GST studies, with an emphasis on recurrent and/or specific breakage patterns (e.g., [19, 21, 29–31, 40–50]).

These studies have highlighted three recurrent patterns as potentially diagnostic of intentional fragmentation: 1) scar(s) of flake removals (especially multiple flake removals), 2) midsection fractures (i.e. roughly parallel fractures resulting in the ‘slicing’ of the object along its width or length) and 3) breakage along multiple axes [19, 21, 50]. In some sites, however, breakage appears as a common phenomenon on different types of GSTs as well as manuports. These broken items are widely distributed within sites, not associated with a specific depositional context (e.g., [47, 51–53]). In such sites, the identification of intentional breakage is particularly challenging, requiring the distinction among various potential agents of breakage and overcoming the issue of equifinality.

Identifying the agent that caused the breakage is complex given that non-anthropic and post-depositional processes may be involved, including weathering, natural fire, and animal trampling, which are known to induce breakage of archaeological implements, even of those made of stone [54–58]. Moreover, when related to anthropic activities, breakage can be accidental, occurring during use or maintenance by exposing the object to fire or dropping it. Multiple flaking is especially diagnostic of intentional breakage as it indicates not only an anthropic origin of breakage, but also that the fragmentation is intentional. Yet in such cases, the intention behind the flaking, which may be reshaping for maintenance, recycling for creating a new tool, or else intentional breakage remains to be established. For these reasons, identifying the agent of breakage and demonstrating intentionality requires the examination of several lines of evidence [59]. In this perspective, this study investigates intentional breakage through the reconstruction of the tool’s life history, by harnessing morphological, technical, 3D, use-wear, and residues as well as contextual analyses (see supplement B in S1 Fig).

**Fig 1:**
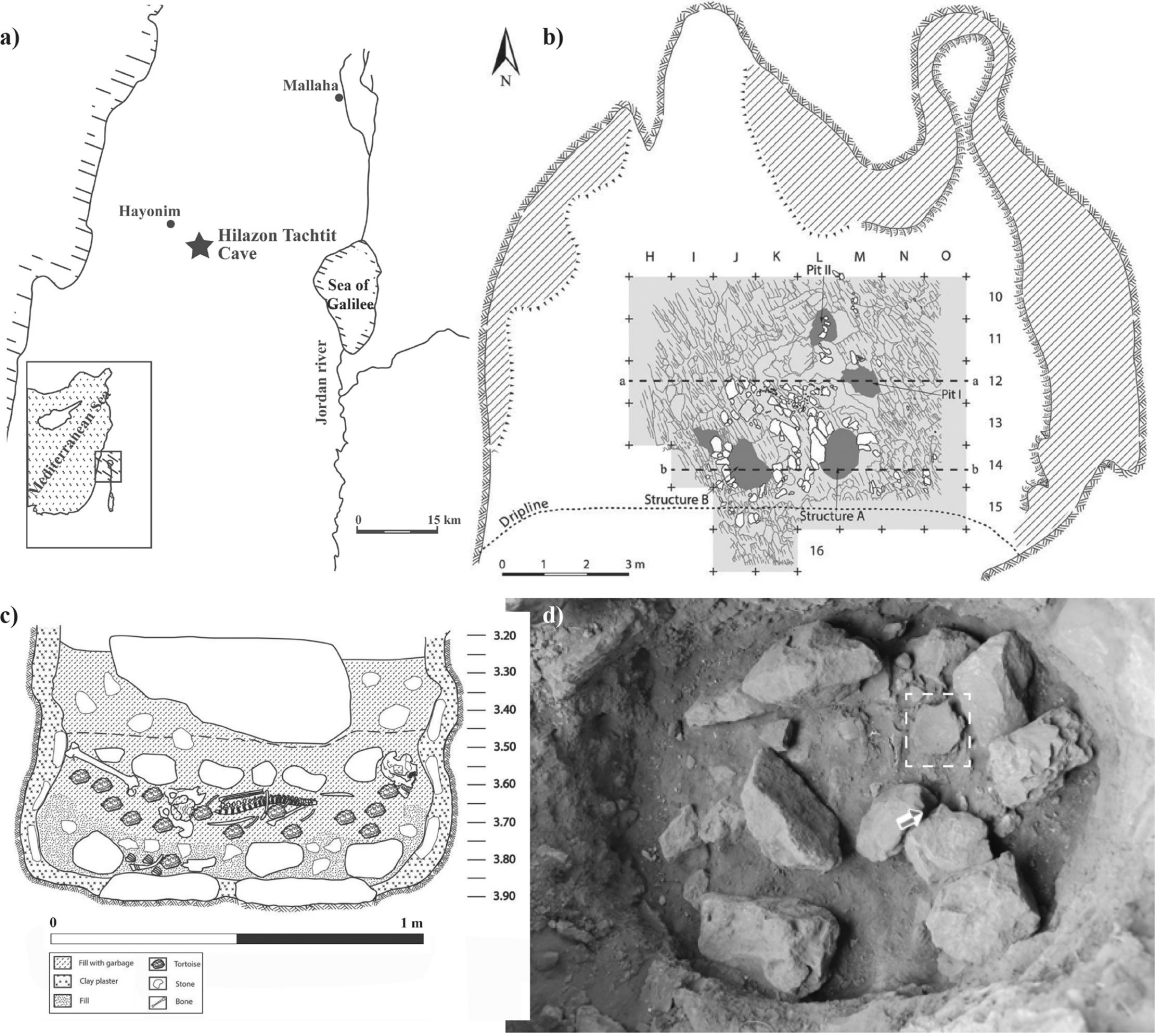
a. Geographic location and general plan of Hilazon Tachtit Cave; b. location of the shaman burial (structure A) (modified from Grosman and Munro, 2016); c. East–west section of the shaman burial; d. GST associated with phase B (modified Grosman and Munro, 2016) of the shaman burial (note that the use surface is facing up).

The broken tool discussed in this paper (catalog number M13c-377, curated at the Hebrew University of Jerusalem) was deposited at Hilazon Tachtit Cave (western Galilee, Southern Levant; Fig 1) in a sealed burial pit dated to 12,000 years ago, during the end of the Natufian culture [24]. The Natufian culture in the Southern Levant has a specific place in time between the Paleolithic way of life and early Neolithic agricultural communities. The Natufian is a well-studied example of new social and economic organization. These include architectural planning (e.g., [60]), complex burial practices (e.g., [24, 30, 61–65]) and large-scale use of GSTs [29, 47, 66–72], among others. Grave goods found in Natufian burials are subjects of ongoing debates regarding the development of social stratification during this period (e.g., [61–62]). Excavations at Hilazon Tachtit cave have furthered our understanding of Natufian social organization and symbolic behaviour, and highlighted the existence of sites primarily devoted to ritual activities. Analyses of the burials at the site also suggested the presence of individuals with specific statuses such as that of a shaman, as well as the occurrence of funerary feasts [24, 64, 73]. The research presented in this paper provides additional insight into Natufian ritual behaviour and, more generally, on practices related to intentional destruction of valuable associated with death-related ceremonies at the end of the Palaeolithic.

## The broken tool: Context of deposition and description

Hilazon Tachtit cave is located in the Lower Galilee (Southern Levant), close to the site of Hayonim Cave, at the top of a 150 m escarpment above the perennial stream of Nahal Hilazon ([74], Fig 1). While the interior surface of the cave is about 100 m^2^, the Natufian occupation corresponds to a ca. 30 m^2^ depression located in the middle of the cave floor. The stratigraphy presents two main units [74]. The upper unit accumulated while the cave was used for overwintering domestic caprines, which most certainly began in the Byzantine period. The Natufian deposit below is dated to the Late Natufian period (see supplement A.2 in S1 Fig). Two small circular structures (ca. 1 m in diameter) and three pits (ca. 0.5 m^2^ each) were uncovered [74]. All of these features contain remains of primary and reopened collective burials (see supplement A.3 in S1 Fig), encompassing a total of 28 individuals [24, 64].

The location of the site on top of a steep, high escarpment, its small size and the large number of interred individuals suggest that the cave had a special ritual function and was devoted to the burial of the dead ([24]; see supplement A in S1 Fig). The types and diversity of flint, bone, GSTs and ornaments are typical for the Natufian period, but GSTs and faunal remains also differ from other sites in aspects that likely relate to the site's function as a burial ground ([73–76], see supplement A.4 in S1 Fig).

The broken tool was found in a unique burial (structure A) that was previously interpreted as a shaman’s grave [24]. The remains correspond to a gracile female (approx. 45 years old and estimated to be 1.5-m tall). The sex was determined primarily based on the shape of the pelvis and the size of the femur [24]. Several skeletal pathologies that accrued during life (vertebral lipping, osteophytes, and heavy erosion of the teeth) indicate that the woman was relatively old. In addition, congenital pathologies such as fusion between the coccyx and the sacrum as well as deformations of the pelvis, lumbar and sacral vertebrae were observed. The burial contained a number of highly unusual grave goods [24, 64] and is unlike any grave found so far in the Natufian, suggesting that the deceased had special status. The tool was interred as part of the burial events [64]. Previous studies have identified six distinct burial phases and reconstructed several aspects of the ritual event [64]. Initially, the bedrock of the cave was cut, forming an oval-shaped pit. The bedrock pit was then plastered with mud and rock slabs were placed within it (Fig 1). Special artifacts, including the broken GST (Fig 1), were placed as a cache between large stones, before the interment of the body. The cache also contained a complete right horn and frontal from a male gazelle, three *Cerastoderma* (a marine bivalve) shells, at least three complete tortoise carapaces, a piece of red ochre, and a chalk fragment [24, 64].

**Fig 2:**
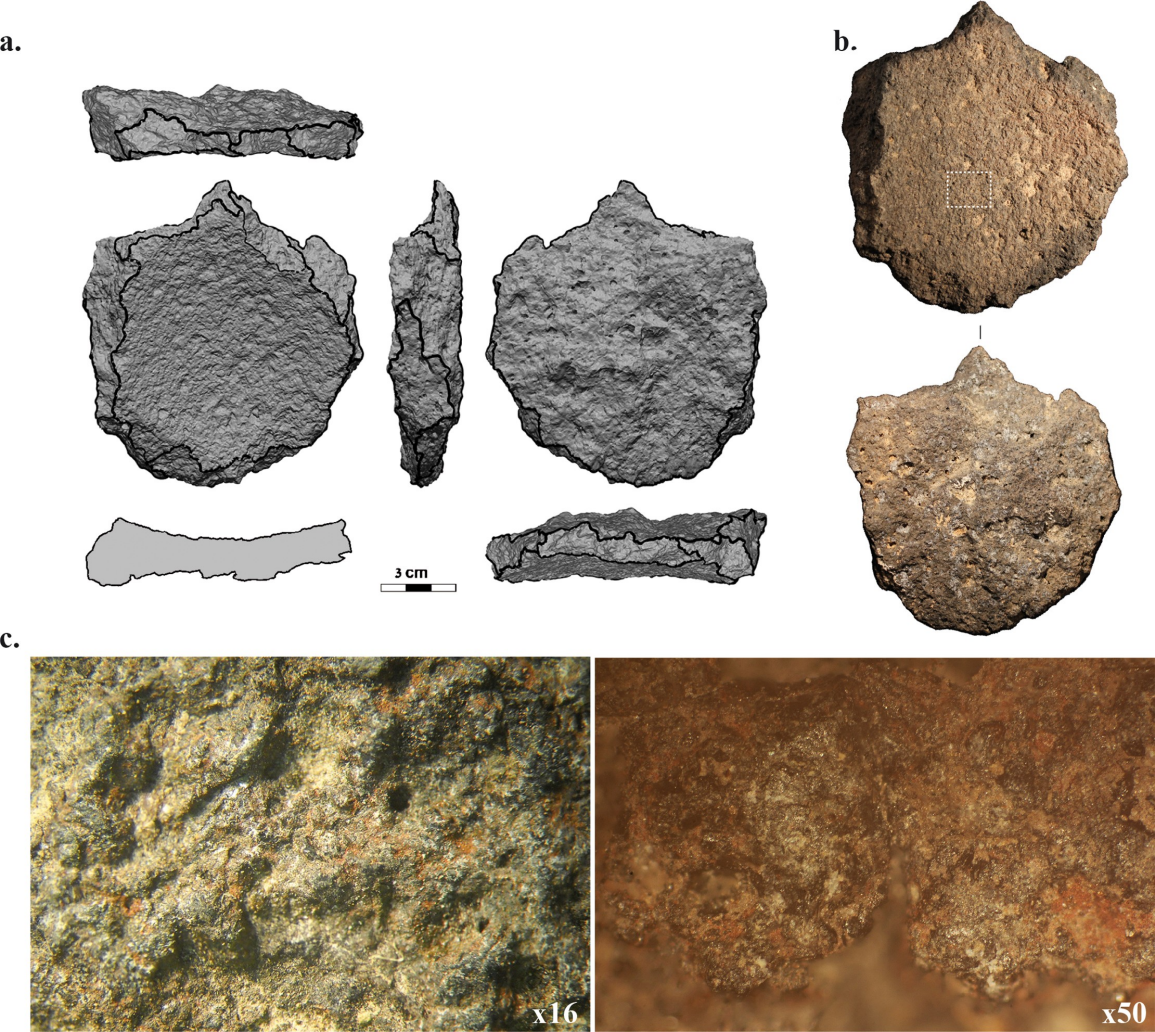
a. 3D scan and b. photograph of the GST (Laure Dubreuil); c. use-wear observed at low and high magnifications on the used surface of the object.

The broken GST is made of fine-grained vesicular basalt and its maximum dimensions reach 11.1 cm in diameter and 2.8 cm in thickness. The fragment has a semi-rounded shape created by various flake removals on its edges and presents a concave working surface. This surface shows reddish coloring, particularly pronounced on one half of the fragment. The reddish coloring does not appear on the bottom or the flaked edges of the GST. The opposite face corresponds to a fracture plane; the surface is irregular and is probably the negative of a single removal. Recognizing characteristics of knapping is difficult on basalt because it tends to break unevenly. Naked eye observations coupled with 3D-scar-segmentation analysis identified a minimum of five flake removal scars on the edges (Fig 2 and see supplement B.1 in S1 Fig). These scars represent flakes that may have been detached before or after the large removal at the base. The precise location of their striking platform cannot be determined. As a result of these flake removals, the GST fragment has an irregular circular contour.

Although it is difficult to determine the original shape of the tool, the convexity of the working surface appears too shallow for a mortar but may fit in the range of grinding-slabs with a marked convexity or else, following Wright’s [71–72] typology, of shallow bowls or platters. Shallow bowls and platters correspond either to mixing tools (also called vessel-mortar) or to serving vessels used as a container rather than for processing matter.

To explore further the typological identification of the broken GST, we carried out a use-wear analysis and investigated whether wear patterns characteristic of pounding or grinding were present on the object. Another goal of the use-wear analysis was to assess whether the fragmentation of the tool could indicate remodelling for recycling.

## Use-wear and residue analysis of the broken tool

Our analysis focuses mainly on the internal working surface, as the rest of the surfaces correspond to fracture planes. Observations combining naked eye with low and high magnification microscopy indicate that the working surface of the GST does not show evidence of grinding. For instance, flat areas resulting from leveling of the microreliefs (creating plateaus) were not observed. Plateaus are commonly associated with grinding during use or with manufacture phases intended to regularize the surface (e.g., [44, 69, 77–79]). Pecking marks are prevalent on the working surface of the GST. According to our reference collection, these marks may relate to a manufacturing phase involving pecking with a hard hammer. A moderate smoothing and rounding of the peaks (asperities of the surface) created by pecking were also observed. This rounding does not seem to be related to tool manufacture, as it does not create a leveling of the surface. The fact that the sides of the peaks are also rounded, and that the rounding process develops into low parts of the microtopography, is consistent with the hypothesis that the rounding is not related to the manufacture of the tool. Instead, the rounding of the peaks can be associated with tool utilization—for instance, for mixing or short-term pounding. The use-wear analysis, therefore, confirms that the tool belongs to the vessel-mortar category and represents a shallow bowl used for mixing or short-term pounding.

At high magnification, a superficial sheen is associated with the smoothing of the peaks and extends into the asperities of the surface (Fig 2). Overall, both the grain alterations observed on the peaks and the sheen characteristics indicate that the matter processed contained a lubricant (most likely water) and an abrasive component (see supplement B.2 in S1 Fig).

A light reddish coloring of the surface was observed, appearing at low magnification as a discontinuous coating over high and low parts of the microrelief (Fig 2). Our analyses suggest either ochre or a natural oxidation of the surface. Because a Fourier Transform Infrared (FTIR) analysis of the reddish coating could not distinguish it from the basalt, identification as ochre seems unlikely. The second option, natural oxidation, may reflect thermal alteration or other processes, and additional research is needed to understand the reddish coloration observed on the working surface of the fragment.

**Fig 3:**
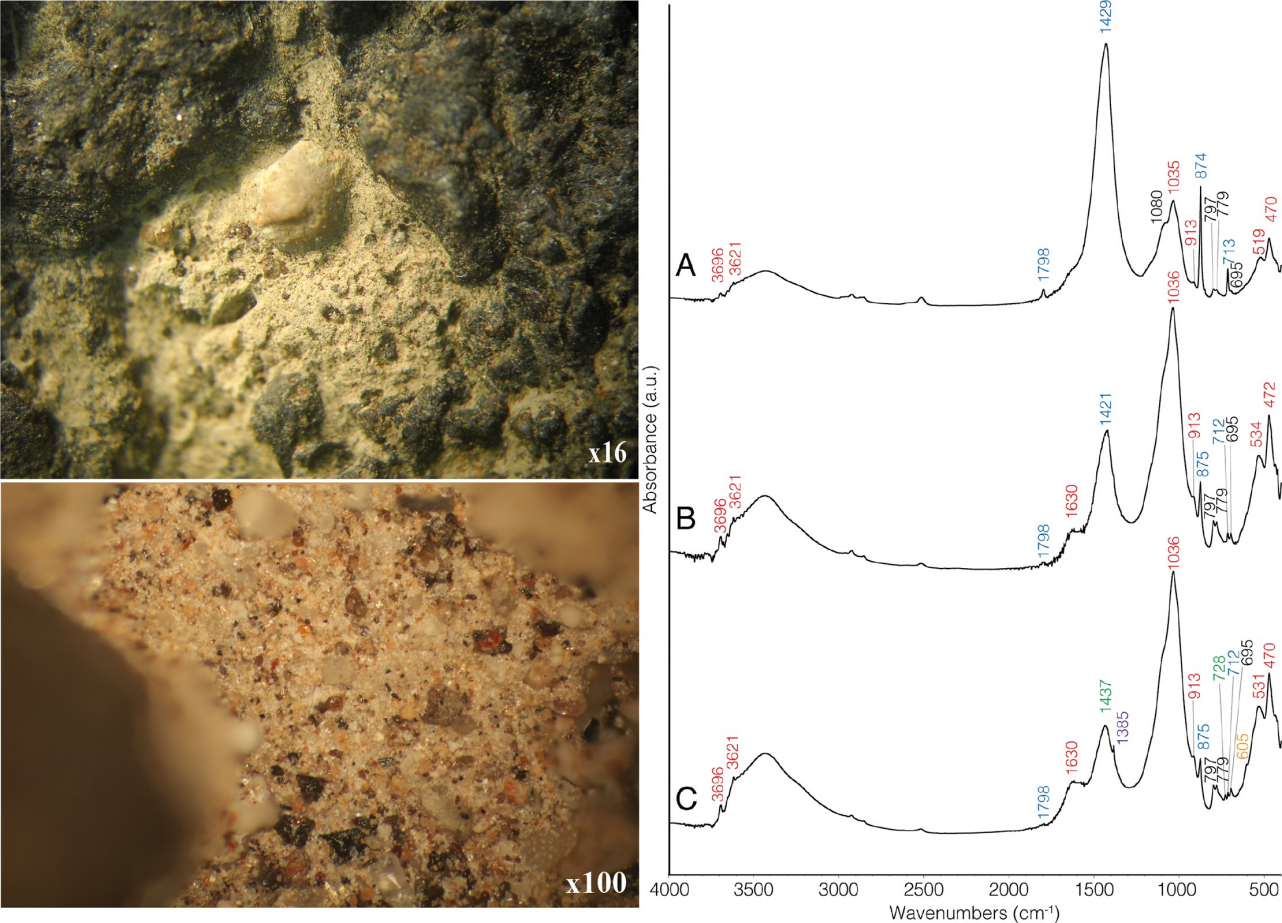
a. whitish residues at low and high magnifications; b. FTIR spectra of (A) the whitish cemented sediment retrieved from the GST’s surface, (B) brownish sediment that was attached to the bottom of the GST, and (C) brownish sediment collected ca. 20 cm below the GST within the burial pit. Absorbance bands typical of clay are marked red; absorbance bands typical of calcite are marked blue; quartz is marked black, dolomite is marked green, phosphate is marked orange and nitratite is marked purple. Note the dominance of clay (unheated) in the sediments, as opposed to the dominance of calcite (pyrogenic) on the GST's surface. Figure prepared with the assistance of Z.C. Dunseth.

The GST was placed in an ultrasonic bath for the extraction of microscopic plant remains. Only a few pollen grains were retrieved. On the working surface of the bowl, some vesicles were filled with a whitish cemented material that remained after the ultrasonic treatment (Fig 3). FTIR analysis revealed that the whitish material is primarily composed of calcite that had been affected by heat, such as wood ash or lime plaster (i.e., pyrogenic calcite, see more in supplement B.3 in S1 Fig). Polarized light microscopy indicated that the whitish cemented material includes micro-charcoal but no other microscopic remains (Supplement B.3, Figure A in S1 Fig). Sediment adhering to the bottom of the tool as well as from the fill 20 cm below the tool were also sampled (samples #2, 3). The mineralogical composition and grain size distribution of these sediments are similar to each other and different from those of the whitish cemented material. Calcite in samples #2 and 3 is less abundant, appearing in the form of rhombs typical of wood ash, associated with grass phytoliths, micro-charcoal and humified vegetal matter (Supplement B.3, Fig A in S1 Fig).

**Fig 4:**
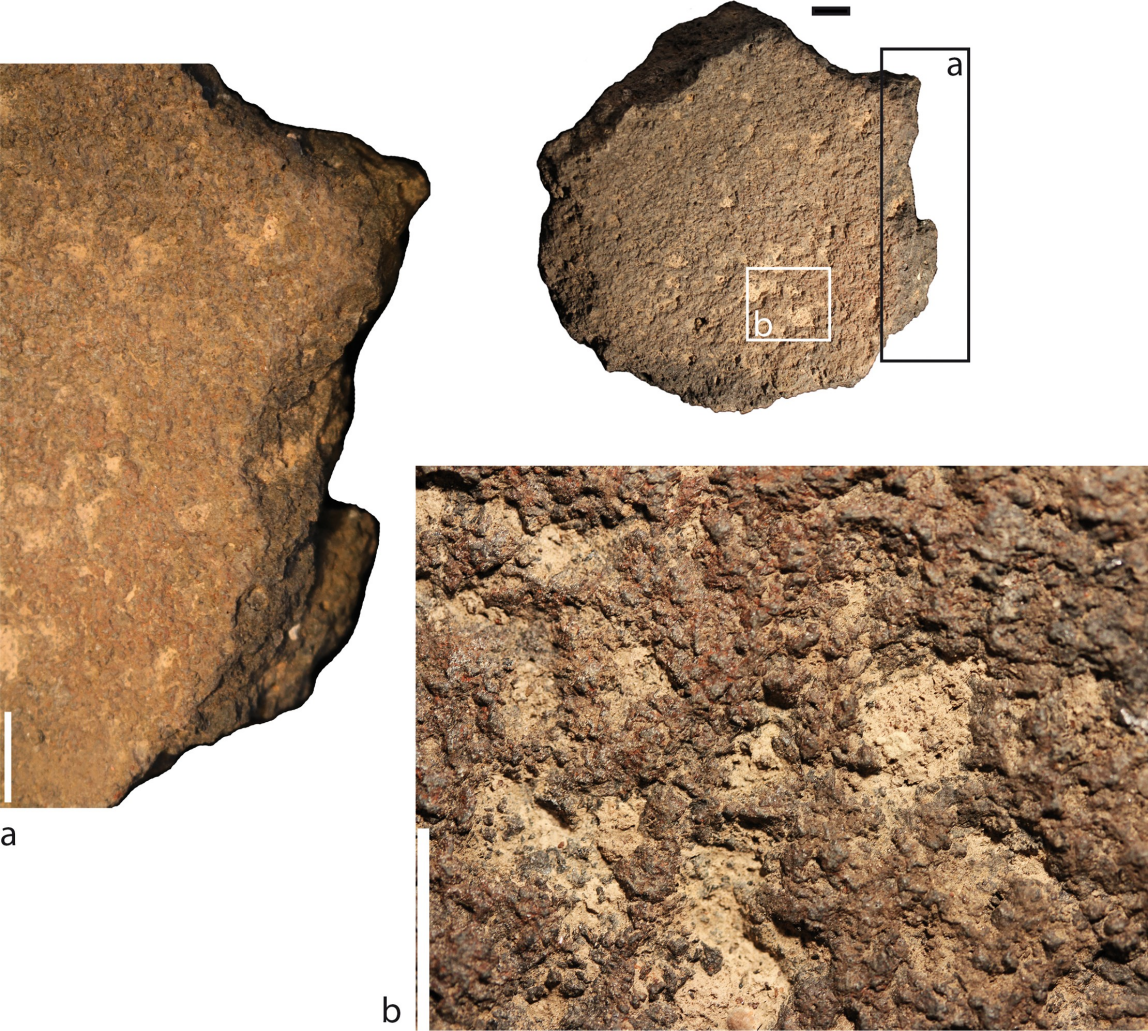
a. flake removal postdating the use-wear that developed on the used surface; b. photograph of the whitish residues overlapping the red coloring of the used surface taken with a SLR 5.3 Mo pixels digital camera with a 55mm macro objective at 1:1. All scale 1 cm.

On the working surface, the whitish cemented residues overlap the red coloration of the surface, implying that the deposition of the residues happened after the reddening of the surface (Fig 4). The indurated nature of the pyrogenic calcite indicates formation associated with water, either originally (during use) or post-depositionally. Use-wear characteristics suggest that the mixing with water was part of the initial processing and not post-depositional. Particularly critical is the fact that the use-wear, red coloring, and residues on the surface are truncated by the flake removals and clearly predate them (Fig 4). Importantly, there is no sign of use or extensive manipulation after the breakage of the bowl.

## The tool’s life history

The analysis of the GST uncovered in structure A bears evidence relating to several stages in its life history:

1. Manufacture: The bowl’s active surface was manufactured by *pecking without intensive leveling*. Only one basalt flake (corresponding to a tool fragment) was retrieved from the entire site, suggesting *off-site manufacturing* of the GSTs uncovered at the cave [76].

2. Utilization: The GST was used for *mixing pyrogenic calcite* (i.e., wood ash or lime plaster) with liquid, probably water. In non-industrial societies, the mixture of ash—or in some cases lime—and water is reportedly associated with food preparation, the production of soap or dye, or used as an insecticide, but also for symbolic purification [80–85]. Interestingly, ‘alkaline ashes’ (ash mixed with water and then filtered, boiled and dried) is mentioned as a component of several different mixtures and is especially used by South American shamans in the preparation of hallucinogenic beverages [16, 18]. Prior to this phase of utilization, the working surface had become red, most likely due to natural oxidation, possibly as a result of burning.

3. Breakage: After its use, the GST was roughly *knapped* into a semi-rounded item. We observed at least 5 knapping scars from different orientations (Fig 2). Because only one basalt flake, originating from a different tool, was found in the entire site [76], the knapping probably took place outside the cave, or the fragments were taken away. There is no evidence for the use or extensive manipulation of the tool after it was broken. Although subsequent flaking conferred an irregular rounded shape, the knapping did not result in the creation of another formal type of tool. Also, the lack of use-wear and residues on the scars clearly indicate that the fragment was not used after the bowl was flaked.

*Intentional breakage* is attested by the presence of multiple knapping scars from different orientations, which rules out mishandling (dropping the tool) or accidental breakage through use.

4. Discard: The intentionally broken shallow bowl is part of a cache of unique items created before the body was interred. This item was *placed at a specific point in time in the burial* sequence of events.

## Discussion

The analysis suggests that the tool was shallow bowl used for mixing pyrogenic calcite, such as wood ash or lime, with water. Importantly, the analysis clearly indicates that this bowl was intentionally broken through multiple flake removals after its utilization and before its deposition in the grave. This sequence of events points to the ritualistic nature of the breakage. This study provides evidence for intentional breakage at the end of the Natufian, a practice that will later became a common phenomenon in human rituals, ranging widely in time and space.

In the ethnographic record, intentional destruction is documented in various contexts, which shows the polysemic nature of this act. For example, breakage is performed for allowing the tool’s spirit to return to the cosmos [9, 19]; to honor the gods or ancestors [5, 19]; for healing rituals or in special community events [5, 13, 85–86]; or to manage conflicts between individuals [8].

In archaeological contexts, various studies have proposed an interpretation for intentional breakage. At Çatalhöyük (Turkey) for instance, Wright [49] suggested that the querns associated with the Neolithic occupation of the site were discarded or destroyed when a house was abandoned. At the Neolithic site of Geleen-Janskamperveld (Netherland), van Gjin and Verbaas [45] proposed a ritual killing of the querns, as an offering to ensure the fertility of the land. In the Holocene record of the Balkans, it is suggested that missing fragments were deposited elsewhere in a process that might have aimed at linking people with places [87–88]. Stroulia and Chondrou [21] offered a similar interpretation for explaining GST fragments dispersion at the Neolithic site of Kremasti (Greece). In general, theoretical discussions on fragmentation highlight the link between intentional breakages, social exchange of fragments linking people to places, and identity construction (e.g., [87–90]). In the Southern Levant, arguments for intentional breakage of GSTs have previously been made for the Natufian [28–30, 85; 91–93] and for earlier periods [28, 29, 91]. For instance, Hayden [86] associated the breakage of large mortars and pestles with funerals and ‘competitive’ feasts. Richter et al. [30] view the intentional breakage and deposition of GST in graves at Shubayqa as part of performances aimed at ‘*dealing with grief and reaffirming social ties*, *identities*, *and roles’* [30:15].

At Hilazon Tachtit, because the GST fragment was deliberately placed into a grave, the reason behind breaking the bowl seems related to burial practices. In ethnographic contexts, grave goods are often reported to be part of the belongings of the deceased (e.g., [94–95]). As previously mentioned, structure A’s burial has been suggested to correspond to a grave of a shaman. It has long been established that shamans, during their life, had personal ritual objects and tools [16, 18]. Preparation of mixtures, as suggested by the functional analysis of the working surface of the bowl, is commonly cited in relation to shamanic ritual activities [16, 18]. Although the information is scarce, ethnographic and historical accounts indicate that the fate of a shaman's toolkit after his/her death is codified and often ritualized and that the most valued tools may be destroyed or passed on to another shaman [16, 18]. Although the reason that motivated the breakage of the bowl found at Hilazon Tachtit may be beyond our reach, possible interpretations include materializing the end of the shaman’s practices, the end of the tool’s life, a sacrifice to prevent retaliation, and the production of ‘symbolic’ tools used in the afterlife.

While some important aspects of the social and symbolic contexts of use and discard of the broken bowl at Hilazon Tachtit remain elusive, this tool, its intentional breakage and disposal in a grave, reflects the cultural tradition in which the deceased operated. Recent studies have allowed for a more comprehensive representation of symbolism and burial practices at the end of the Natufian showing the existence of sites devoted to funerary activities [24, 96–98], of feasting [72, 97] and the use of flowers in burial rituals [63]. The broken bowl found in a burial pit at Hilazon Tachtit demonstrates that intentional breakage was also part of burial practices. Dating to the same period, Raqefet Cave and Nahal Oren burial grounds also provide evidence for the association of ‘perforated’ or fragmented mortars with burials [29, 92, 98]. At Shubaqay 1, Richter et al. [30] also underline the high number of broken GST associated with burials attributed to the Early and Late Natufian occupations of the site. Additional in-depth contextual analysis and reconstruction of the life-history of GSTs deposited in graves during the Natufian should provide valuable data for understanding burial practices and the symbolic dimension of GSTs in the transition from foraging to farming.

In general, breakage patterns observed in Natufian GST assemblages suggest that intentional fragmentation was a broader phenomenon, beyond the specific context of burial practices, and was likely associated with various meanings [50]. This organized tool breakage provides an important window into Natufian ritual practices during the critical period that materialized the transformation of these societies into agricultural communities.

## Supporting information

S1 Fig Microphotographs of sediment spreads of (a) the whitish cemented sediment retrieved from the GST’s surface, and (b) the brownish sediment attached to the bottom of the GST. Note the difference in grain-size distribution between the two sediments (images taken at the same magnification). Black particles in (a), marked as "1," represent charred particles. Particles in (b) are wood ash crystals (1), a grass phytolith (2), charred organic matter (3), and humified organic matter (4).
(DOCX)
