# Supplementary material for: Evidence of ritual breakage of a ground stone tool at the Late Natufian site of Hilazon Tachtit cave (12,000 years ago)
Source: PLoS One. 2019 Oct 16;14(10):e0223370. doi: 10.1371/journal.pone.0223370 (PMC6795415; doi:10.1371/journal.pone.0223370)
Supplement: S1 Fig — Microphotographs of sediment spreads of (a) the whitish cemented sediment retrieved from the GST’s surface, and (b) the brownish sediment attached to the bottom of the GST. Note the difference in grain-size distribution between the two sediments (images taken at the same magnification). Black particles in (a), marked as "1," represent charred particles. Particles in (b) are wood ash crystals (1), a grass phytolith (2), charred organic matter (3), and humified organic matter (4). (DOCX) [file pone.0223370.s001.docx]

**Supplementary Information 1**

***A. Site and Context***

**A.1)** Location and formation

Hilazon Tachtit cave was formed through the dissolution of a hard limestone shelf belonging to the Upper Cenomanian Yanuch Formation (Frumkin and Flischhendler 2005). The cave itself is small (ca. 100m^2^) and lacks a front terrace (Grosman, 2003).

**A.2)** Stratigraphy

The stratigraphy of the deposit comprised two main units: - a thick upper layer composed of ashes and goat dung resulting from the use of the cave for overwintering domestic caprines, which most certainly began in the Byzantine period (as indicated by the presence of a few pottery fragments); - a 1.20 m thick Natufian layer. Absolute dating indicates an occupation during the later stage of the Natufian period (Grosman and Munro 2007): 12,400–12,000 calBP (RTT 3760, RTT 4592, RTT 4593). The Late Natufian deposit is homogeneous overall, disturbed in one locality on its upper surface (top 20 cm) by historical digging. However, the Natufian burial features were not disturbed.

**A.3)** Structures and burials

The site consisted of two circular structures and three pits. The structures are small (ca. 1 m in diameter) (Grosman and Munro 2007). Next to the structures are 3 small burial pits (c.a. 0.5 m^2^ each) that occupy an area of 5 m^2^ to a depth of 80 cm.

Three primary burials were found inside and between the two structures while the pits contained remains of collective burials. These collective burials represent the remnants of individuals who were originally interred in the pits but were later disturbed when the graves were reopened and the long bones and skulls were removed. One of the three primary burials, a young adult buried in a flexed position associated with the scattered bones of a newborn, was found at the top of the Natufian sequence and was partially disturbed by later activities. The other two primary burials belong to an earlier phase and are interred in graves within structures. In total, 28 individuals were buried in the cave.

**A.4)** Material culture and faunal remains

The material culture, especially the lithic assemblage, confirmed an attribution to the Late Natufian phase. Forty per cent of the Hilazon Tachtit tool assemblage is comprised of microliths. The geometric microlith category is dominated by lunates (88%). They are short and abruptly backed, characteristics of the Late Natufian period (Grosman and Munro 2007). The ground stone assemblage is made of 63 tools (and 51 manuports) and comprises grinding and pounding implements, as frequently found in Natufian sites. However, rather uncommon types of ground stone tools were uncovered at the site, including a fragment of a large vessel, a combined handstone / grinding-slab implement (Dubreuil and Grosman 2013).

The faunal assemblage is divided into three major taxonomic groups including ungulates (35.7%), carnivores (3.9%) and small game (60.4%; Grosman and Munro, 2007). Gazelle (30.0%) and tortoise (45.4%) dominate the assemblage. Similar proportions are found in other Late Natufian assemblages in the Mediterranean zone, such as Hayonim Terrace and Hayonim Cave (Munro 2004). The presence in two structures of unprecedented density and quantity of aurochs (wild cattle; *Bos primigenius*) and Mediterranean spur-thighed tortoise (*Testudo graeca*) suggest that some of the faunal remains were accumulated through feasting events in relation with the burial practices (Munro and Grosman 2010).

***B. 3D, use-wear and residue analysis***

The methods used in this research combine morphological studies, typological classification, and technological, use-wear and residue analyses. The objective of the study was to better understand the life history of the tool, including its manufacturing process, use, and possible episodes of reshaping and recycling.

The shape and breakage patterns analysis incorporates 3D modeling. The methods and primary results of the 3D, use-wear, and residue analyses are presented below.

***B.1) 3D Analysis***

The GST was 3D-scanned at high resolution in the Computational Archaeological Laboratory of the Institute of Archaeology (Hebrew University of Jerusalem) and analyzed using the software developed at this laboratory (Grosman 2016; Grosman, Smikt, and Smilansky 2008; Grosman et al. 2014; Karasik and Smilansky 2008; Richardson et al. 2014). As flaking can be difficult to identify on coarse-grained rocks such as basalt, the scar-segmentation feature on the 3D model was combined with visual observation to assess the flake removals present on the object. By segmenting scars on the model through the estimation of maximum principal curvatures on the tool surface, and after clustering, the scanner can detect the ridge lines, i.e., the borders of each removal scar (see Richardson et al. 2014 for a detailed explanation).

***B.2) Use-wear analysis***

*B.2.1) Materials and Methods*

Use-wear analysis followed a well-established protocol detailed in several papers (e.g., Adams et al. 2009; Dubreuil et al. 2015). The reference collection used for interpreting the use-wear patterns comprises mostly basalt implements and includes grinding slabs, handstones and abraders (Dubreuil 2004), as well as mortar and pestle–like tools from an ongoing experimental program exploring the wear associated with different manufacture techniques, as well as the processing of various types of mineral, vegetal, and animal matter.

In this study, the entire tool surface was examined at various magnifications, including naked eye and low magnification observations with a stereomicroscope (using a ZEISS binocular from 6X to 40X) and high magnification observations with a metallographic microscope (using an Olympus microscope with 50X to 500X objectives, equipped with differential interference contrast). The analysis draws on the comparison between distinctive parts of the tool fragment, encompassing the used, inner convex surface; the surfaces of the scar removals; and the opposite face of the used surface.

*B.2.2) Results*

The appearance of both the scar removals and their opposite surface reflects a natural fracture plan of basaltic rocks, and lack conspicuous evidence of use or alteration. The scar removals, however, do show different patina from each other.

The used surface is significantly different from the other parts of the tool and displays wear patterns which can be characterized as follows:

– *Naked eye examinations*: The surface is relatively regular (low amplitude between the high and low parts of the micro-relief, in comparison to the surface of a fracture plan), but rough (not flat and smooth). There are no plateaus, which correspond to zones of intense leveling of the highest part of the micro-relief creating a localized flat surface. Plateaus are commonly associated with grinding action or manufacture phases intended to regularize the surface. Pecking marks are common. At this scale of observation, the wear characteristics (type and distribution) observed on the tool’s active surface can be associated, according to our reference collection, with the pecking of the surface with a hard hammer (Dubreuil, 2002, 2004). A reddish coloring of the used surface is observed and is particularly pronounced on one half of the fragment.

– *Low magnification observations*: The picks , which correspond to the high topography of the relief created by the pecking of the surface, present moderate smoothing and rounding, indicating they underwent further modifications after the pecking of the surface. The smoothing is not restricted to the summits of the picks but is also present on their edges, the distribution of this wear pattern thus showing high amplitude. Grain removals and grain leveling are also observed on the picks. These wear patterns are associated with a moderate sheen.

The limits of the area with a more pronounced reddish coloration are diffuse. The reddening is more intense on one part, nevertheless it affects the entire surface. The reddish coloration does not represent an alteration of the phenocrystals naturally present in the rock, as sometimes observed on basalt. Despite intense cleaning, a few concavities remain filled with a whitish sedimentary material that overlaps the reddish coloration of the surface when present.

– *High magnification observations*: The presence of specific grain alteration on the picks, encompassing leveling and edge smoothing, is confirmed. Large grains with micro-fractures are also observed. High magnification observations allow further characterization of the sheen present on the picks. The sheen appears to be extensive on the surface, showing high amplitude in its distribution across the micro-relief. The sheen can be described as translucent, superficial, and generally fluid. However, some flat and oriented components are also observed (i.e., not displaying clear striation but showing a preferential direction) . It should be noted that no conspicuous variation in wear characteristics was found between the area where the reddish coloration is pronounced and the rest of the surface.

*B.2.3) Interpretation*

The presence of picks can be related to a manufacturing phase involving surface pecking. The absence of plateaus, the rounding and smoothing of the picks, the alterations of the grains, and the presence of a sheen suggest utilization associated with mixing, or a light pounding type of motion, rather than forceful strokes or a highly abrasive action such as pounding or grinding. Therefore, the present analysis suggests that the tool was a mortar/vessel (platter or shallow bowl-mortar) used for mixing and/or light pounding. The use-wear characteristics also indicate that the matter processed contained a lubricant, most likely in the form of water, as no clear wear patterns indicating the presence of grease was found. Some characteristics of the sheen and the grain’s alteration also suggest that the processed matter had an abrasive component. The mixing or light pounding may be related to the deposition of the whitish material still found in some concavities after deep cleaning.

***B.3) Residue analysis***

*B.3.1) Materials and methods*

A microarchaeological analysis was carried out on three sediment samples:

1. The hard-gray fine sediment that fills pores in the basalt bowl
2. The brownish-gray sediment attached to the bottom of the bowl
3. The brownish-gray sediment collected from the fill 20 cm below the bowl

Sample #1 is thought to be associated with the use of the bowl, while samples #2 and 3 were used as controls. It is expected that a difference between samples #1 and #2–3 will support the former being related to usage.

Analyses included Fourier Transform Infrared spectroscopy (FTIR), which allows identification of mineral components in sediments and enables determination of whether certain minerals have been heated in the past (Weiner 2010). FTIR analysis used the conventional KBr method; about 0.1mg of the sample was ground and mixed with ca. 1mg KBr and pressed into a transparent pellet. The latter was introduced into a Thermo Scientific Nicolet iS5 spectrometer and analyzed between 4000 and 400 cm^-1^ at 4cm^-1^ resolution. The resulting spectra were assessed in reference to interpretational guidelines given in Berna et al. (2007) for clay minerals, and Regev et al. (2010) for the mineral calcite.

Additionally, the sediment samples were observed in grain mounts using petrographic microscopy. Grain mounts were prepared from ca. 1mg sediment sample on a microscope slide, immersed in a few drops of biological glue (Entellan, Merck) and covered by a coverslip. The observation was conducted at 100X, 200X and 400X in plane and crossed polarized light, using a Nikon Eclipse 50i POL microscope.

*B.3.2) Results*

All three sediment samples are composed of calcite, clay, and quartz. A major difference is the relative amount of clay and calcite, with sample #1 (from the bowl’s active surface) being predominantly composed of calcite, while samples #2 and 3 (controls) are chiefly composed of clay (Main text Figure 3b).

In all three samples, the clay component includes absorbance bands of hydroxyls (at 3696 and 3621 cm^-1^) indicating that the clay component has not been heated above 500°C (based on criteria given in Berna et al. 2007). Analysis of peak heights and widths of the calcite component (normalized ν2 height is 406; normalized ν4 height is 103), suggests the calcite is pyrogenic and may be related to either wood ash or lime plaster (following Regev et al. 2010). In comparison, the calcite in the control samples (with normalized ν2 heights at 326 and 276 and normalized ν4 heights at 72 and 66, for samples #2 and #3 respectively), suggest a geogenic source (following Regev et al. 2010).

Other components identified through FTIR spectroscopy include the mineral nitratite (NaNO_3_), found in sample #3, that often forms from water evaporation associated with degradation of organic matter (Shahack-Gross et al. 2004). The spectrum of this same sample also includes absorbance bands typical of phosphate compounds (at 605 cm^-1^) and dolomite (at 1437 and 728 cm^-1^). Phosphate compounds may originate from degradation of organic matter (Shahack-Gross et al. 2004) while the dolomite is probably from bedrock.

Microscopic observations show that the calcite in sample #1 is composed of microscopic particles that do not appear in the forms typical to wood ash (i.e., the so-called pseudomorphs of calcite after calcium-oxalates; Shahack-Gross and Ayalon 2013); however, micro-charcoal fragments are abundant (SI Figure A).


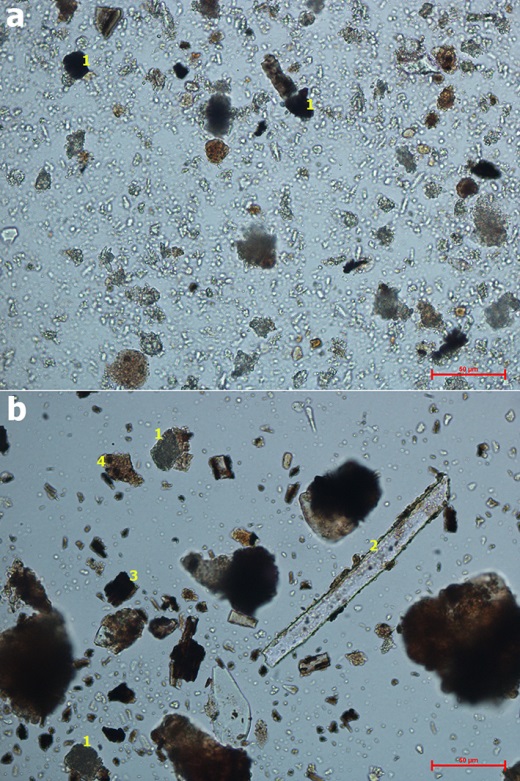


**Fig. A**.

*B.3.3) Discussion*

The analyses conducted reveal that sample #1 (from the GST’s active surface) has a different mineralogical composition compared to samples #2 and 3. This difference suggests that while sample #1 indeed relates to the use of the GST, both samples #2 and 3 appear to represent the sediment in the grave’s fill, composed primarily from unheated clay, some quartz, and calcite. The calcite comes from both a geogenic origin and wood ash.

Sample #1 is dominated by pyrogenic calcite associated with micro-charcoal, and no phytoliths. Such an association is expected in either wood ash or lime plaster. While wood ash is expected to include typical pseudomorphs, lime plaster is typified by extremely small crystallites. Thus, based on its microscopic appearance, the pyrogenic calcite on the GST’s surface most resembles lime (as ash pseudomorphs are absent). We note, however, that wood ash heated to very high temperatures (>800°C) will also transform into lime. Although we cannot determine with confidence whether the pyrogenic calcite in the sample comes from ash or lime, its cemented appearance indicates induration by water. The latter may have been associated with the pyrogenic calcite either during human activity (purposeful mixing with water) or post-depositionally (water dripping in the cave).

*B.3.4) Conclusion*

The sediment within the pores of the basalt bowl has been identified as cemented pyrogenic calcite originating from either wood ash or lime.

**References**

Adams J, Delgado S, Dubreuil L, Hamon C, Plisson H, Risch R (2009) Functional Analysis of Macro-Lithic Artifacts. *Non-Flint Raw Material Use in Prehistory: Old Prejudices and New Directions,* eds Sternke L, Costa LJ, Eigeland L (Archaeopress 1939, Oxford), pp. 43–66.

Berna F, Behar A, Shahack-Gross R, Berg J, Boaretto E, Gilboa A, Sharon I, Shalev S, Shilstein S, Yahalom-Mack N, Zorn J.R, Weiner S (2007) Sediments exposed to high temperatures: reconstructing pyrotechnological processes in Late Bronze and Iron Age strata at Tel Dor (Israel). *J Archaeol Sci* 34: 358–373.

Dubreuil L (2002) *Etude Fonctionnelle Des Outils de Broyage Natoufiens : Nouvelles Perspectives Sur l’émergence de l’Agriculture Au Proche-Orient*. Unpublished Ph.D. Thesis (Université de Bordeaux I).

Dubreuil L (2004) Long-term trends in Natufian subsistence: A use-wear analysis of ground stone tools. *J Archaeol Sci* 31:1613–1629.

Dubreuil L, Savage D, Delgado-Raack S, Plisson H, Stephenson B, de la Torre I (2015) Current analytical frameworks for studies of use–wear on ground stone tools. *Use-Wear and Residue Analysis in Archaeology*, eds Marreiros JM, Gibaja Bao JF, Ferreira Bicho N (Springer International Publishing, Cham), pp. 105–158.

Dubreuil L, Savage D (2014) Ground stones: A synthesis of the use-wear approach. *J Archaeol Sci* (48):139–53.

Frumkin A, Fischhendler I (2005) Morphometry and distribution of isolated caves as a guide for phreatic and confined paleohydrological conditions. *Geomorphology* 67:457–471.

Grosman L (2003) Preserving Cultural Traditions in a Period of Instability: The Late Natufian of the Hilly Mediterranean Zone. *Curr Anthro* 44(4): 571–80.

Grosman L, Munro N (2007) The Sacred and the Mundane: Domestic Activities at a Late Natufian Burial Site in the Levant. *Before Farming* 4: 1–14.

Grosman L, Munro N, Belfer-Cohen, A (2008) A 12,000-year-old shaman burial from the Southern Levant (Israel). *PNAS* 105 (46):17665–17669.

Grosman L, Smikt O, Smilansky U (2008) On the application of 3-D scanning technology for the documentation and typology of lithic artifacts. *J Archaeol Sci* 35 (12):3101-3110.

Grosman L,  Karasik A, Harush O, Smilansky U (2014) Archaeology in three dimensions: Computer-based methods in archaeological research. *Journal of Eastern Mediterranean Archaeology and Heritage Studies* 2 (1):48-64.

Grosman L (2016) Reaching the point of no return: The computational revolution in Archaeology. *Annual Review of Anthropology* 45:129-145.

Grosman L, Munro N (2016) A Natufian Ritual Event. *Curr. Anthropol*. 57(3):311–331.

Karasik A, Smilansky U (2008) 3D scanning technology as a standard archaeological tool for pottery analysis: Practice and theory. *J Archaeol Sci* 35 (5):1148-1168.

Munro N, Grosman L (2010) Early evidence (ca. 12,000 B.P.) for feasting at a burial cave in Israel. *PNAS* 107(35):15362–15366.

Regev L, Poduska K.M, Addadi L, Weiner S, and Boaretto E (2010) Distinguishing between calcites formed by different mechanisms using infrared spectrometry: archaeological applications. *J Archaeol Sci* 37: 3022–3029.

Richardson E, Grosman L, Smilansky U, Werman M (2014) Extracting scar and ridge features from 3D-scanned lithic artifacts. *Archaeology in the Digital Era: Papers from the 40th Annual Conference of Computer Applications and Quantitative Methods in Archaeology (CAA), Southampton,*26–29 March 2012, eds Earl G, Sly T, Chrysanthi A, Murrieta-Flores P, Papadopoulos C, Ramonowska I, Wheatley D, pp. 83–92. (Amsterdam University Press, Amsterdam).

Shahack-Gross R, Ayalon A (2013) Stable carbon and oxygen isotopic compositions of wood ash: an experimental study with archaeological implications. *J Archaeol Sci* 40: 570–578.

Shahack-Gross R, Berna F, Karkanas P, Weiner S (2004). Bat guano and preservation of archaeological remains in cave sites. *J Archaeol Sci* 31:1259-1272.

Weiner S (2010) *Microarchaeology: Beyond the Visible Archaeological Record*. Cambridge, UK.
